# Validation of Walking Speed Estimation from Trunk Mounted Accelerometers for a Range of Walking Speeds

**DOI:** 10.3390/s21051854

**Published:** 2021-03-06

**Authors:** Sietse M. Rispens, Lieke G. E. Cox, Andreas Ejupi, Kim Delbaere, Janneke Annegarn, Alberto G. Bonomi

**Affiliations:** 1 Philips Research, Remote Patient Management and Chronic Care Department, High Tech Campus 34, 5656 AE Eindhoven, The Netherlands; sietserispens@hotmail.com (S.M.R.); andreas.ejupi@philips.com (A.E.); janneke.annegarn@philips.com (J.A.); 2 Philips Research, Patient Care & Monitoring Department, High Tech Campus 34, 5656 AE Eindhoven, The Netherlands; alberto.bonomi@philips.com; 3 Falls, Balance and Injury Research Centre, Neuroscience Research Australia, University of New South Wales, Sydney, NSW 2031, Australia; k.delbaere@neura.edu.au; 4 School of Population Health, University of New South Wales, Sydney, NSW 2033, Australia

**Keywords:** slow walking, speed estimation, inverted pendulum, accelerometer

## Abstract

Walking speed is a strong indicator of the health status of older people and patients. Using algorithms, the walking speed can be estimated from wearable accelerometers, which enables minimally obtrusive (longitudinal) monitoring. We evaluated the performance of two algorithms, the inverted pendulum (IP) algorithm, and a novel adaptation correcting for lateral step movement, which aimed to improve accuracy during slow walking. To evaluate robustness, we gathered data from different groups (healthy adults, elderly, and elderly patients) of volunteers (n = 159) walking under various conditions (over ground, treadmill, using walking aids) at a broad range of speeds (0.11–1.93 m/s). Both of the algorithms showed good agreement with the reference values and similar root-mean-square errors (RMSEs) for walking speeds ≥0.5 m/s, which ranged from 0.09–0.16 m/s for the different positions, in line with the results from others. However, for slower walking, RMSEs were significantly better for the new method (0.06–0.09 m/s versus 0.15–0.19 m/s). Pearson correlation improved for speeds <0.5 m/s (from 0.67–0.72 to 0.73–0.82) as well as higher speeds (0.87–0.97 to 0.90–0.98) with the new method. Overall, we found that IP(-based) walking speed estimation proved to be applicable for a variety of wearing positions, conditions and speeds, indicating its potential value for health assessment applications.

## 1. Introduction

Walking speed, which is also often referred to as gait speed, is a strong indicator of the health status of older people. In this population, walking speed is positively associated with survival [1] and negatively with fall incidence [2,3]. In addition, it predicts adverse outcomes in community-dwelling older people [4], as well as hospitalization and dependence [3]. In hospital settings, walking speed is used for the assessment of acutely ill older people [5], and six-minute walk tests (6MWTs) are common for the objective assessment of functional exercise capacity for the management of patients with moderate-to-severe pulmonary disease [6].

Assessing walking speed as a clinical parameter typically requires a visit, either of a patient to the clinic or of a professional to the patient. An accurate estimation of walking speed using a wearable sensor could be a convenient alternative for these visits. In addition, it could provide information regarding walking speed throughout the day as well as changes over longer periods of time. Zijlstra and Hof introduced an elegant method to estimate walking speed from a wearable accelerometer, based on the inverted pendulum (IP) model for walking [7]. This method determines the height changes by double integration of vertical accelerations that were collected at the lower back. Assuming a circular movement of the pelvis with a radius equal to the leg length, one can derive step lengths from the height changes and leg length using geometric principles. An advantage of the IP model when compared to abstraction models, as discussed, for example, by Yang and Li [8], is that it is less dependent on training data, which typically misses very slow walking and patient data. Therefore, a biomechanical model may extrapolate better to these conditions. Other human gait or direct integration walking speed estimation methods [8] are based on lower limb attachment of sensors, whereas we are particularly interested in trunk locations.

The IP method has been developed and successfully used for a sensor location on the lower back [7]. However, in home and hospital settings, other sensor locations could be preferred, such as a pendant, which is similar to certain personal emergency response systems, or the chest for simultaneous heart rate and respiratory rate monitoring. Furthermore, the IP method was only assessed for moderate to fast speeds of 0.5 m/s and higher, while (frail) older people and, especially patients, may ambulate at speeds below 0.5 m/s [9].

In this study, we aimed to assess the performance of the IP method for pendant and chest wearing positions, for speeds ranging from below 0.2 m/s to above 1.5 m/s. In addition, we aimed to assess the performance of an adapted IP method that we developed to improve estimations, especially for low walking speeds.

## 2. Materials and Methods

### 2.1. Studies

**Figure 1:**
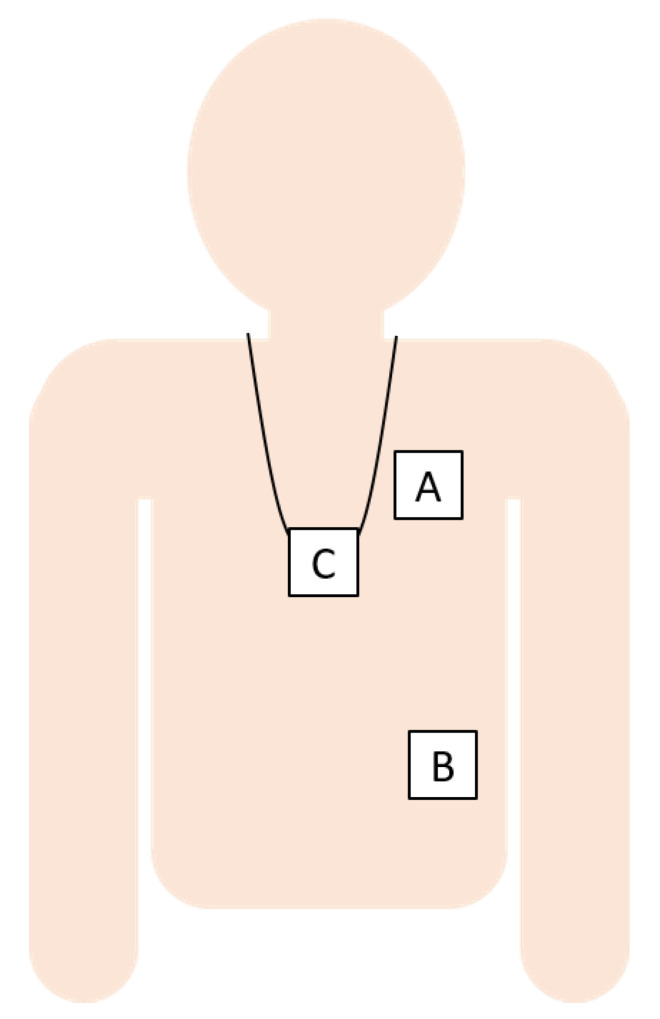
The sensor locations were the left chest (**A**), left lower rib (**B**), and pendant (**C**).

We used data from four studies, which, together, provide data for the three wearing locations, i.e., pendant, left-side of the chest, and left lower rib (see Figure 1), and for various populations and walking conditions. Each of the studies complied with the principles laid down in the Declaration of Helsinki and all of the studies have been approved by the appropriate ethical committees that are related to the institution(s) in which they were performed. All of the participants provided informed consent. In total, 159 volunteers participated in the four studies, which resulted in a dataset with sufficient variation in walking speeds and conditions for all three wearing positions for algorithm performance assessment. Two types of acceleration sensors were used: GENEActiv, referred to here as “GA”, from Activinsights, 43 × 40 × 13 mm, recording three-dimensional (3D) accelerations at sampling frequency of 100 Hz, with +/−8 g range at 3.9 mg resolution, and a pendant device (Philips Research, Eindhoven, The Netherlands, approximate size: 39 × 12 × 63 mm), referred to here as “SMM” (more details and prior use have been described, for example, by Saporito et al. [10]), with a triaxial accelerometer (Analog Devices ADXL362, range of ±8 G, sampling rate 50 Hz).

The first study, referred to here as “Young”, was carried out at Philips Research, Eindhoven, The Netherlands. Employees (18–65 years) without movement disorders that volunteered walked 100 steps over ground six times; at normal, slow, medium and irregular pace, and as asymmetric conditions walking with a weight in one hand and with an eraser fixed under one shoe. Three-dimensional (3D) accelerations were recorded at pendant (SMM, worn over the clothes), left chest (GA, worn in a belt strapped around the chest), and left lower rib (GA, attached to the skin using adhesive tape) locations. The reference speed was obtained by taking the distance from a laser distance meter at start and end of each condition, and while taking the time from the video annotations. 

The second study, referred to here as “Out-Patients”, was carried out at Maxima Medical Center, Veldhoven, The Netherlands. Elderly patients presenting at the Outpatient Department of Internal Medicine were recruited for participation in the study. The inclusion criteria were age above 65 years, patients not using walking aids, not having allergies for leather, and not carrying any pacemaker device. The volunteers were asked to walk at normal pace for approximately one minute, back and forth over a 20 m distance, and finishing at one of the two turning points. 3D accelerations were recorded at pendant (SMM, worn over the clothes) and left chest (GA worn in a belt strapped around the chest) locations. The reference speed was obtained by video annotating time and distance. 

The third study, referred to here as “Simulated Hospital”, was carried out at Philips Research, Eindhoven, The Netherlands. Adult (18–65 year) volunteers without movement disorders or allergies or sensitivities to leather, stainless steel, or medical grade adhesives were asked to walk on a treadmill at different speeds for two minutes, to simulate patient walking over ground with a walker, crutches, a rollator, and a pole for 1 min. each, and to perform a 6-min. walk test for maximum distance, referred to here as “Treadmill”, “Walking Aid”, and “6MWT”, respectively. 3D accelerations were recorded at pendant (GA, worn over the clothes), left chest (GA, attached with medical tape to the torso), and left lower rib (GA, attached with medical tape to the torso) locations. The reference speed for walking at the treadmill was taken as the speed that was indicated by the treadmill. The over ground walking activities were carried out indoors on a marked 30 m course and the distance was measured by the researcher at the end of each activity (timed using a stop watch), with video present to check annotations, if needed. More details of this data collection study can be found in the paper of Fridriksdottir and Bonomi [11].

The fourth study, referred to here as “Community”, was carried out under the iStoppFalls project [12]. Older (≥ 65 years), medically stable volunteers living in the community and not suffering from major cognitive impairment or neurogenerative disease were assessed in the laboratory and then walked a distance of 14 m twice at their comfortable normal walking speed, while wearing an SMM around at the pendant location under their clothes. The middle 10 m were timed with a stopwatch. To enable this, there were tape lines on the floor at 0, 2, 12, and 14 m, and recording started when the person made first contact with the 2m line and it was stopped when the person made first contact with 12 m line. Table 1 presents the demographics of the four studies. 

### 2.2. Walk Data Selection

For the Young, Out-Patients, and Simulated Hospital studies, the data were video-annotated, and the walking acceleration signals were selected after synchronization. For treadmill walking, the first 10 s and the last 5 s were removed, in order to avoid the starting and stopping of the treadmill to cause the speeds to be different from the annotated ones. For the Community study, accelerations were recorded per 14 m walk. To get only the accelerations of the middle 10 m walking, we searched the acceleration signal for the segment with maximum power, where the duration of the segment had to be equal to the 10 m time. 

### 2.3. Estimation of Walking Speed

The IP method for speed estimation assumes that, during the single support phase, the body center of mass (CoM) follows a circular trajectory with a radius *l* equal to leg length, thereby leading to CoM height changes that are directly related to step lengths *s* as $s=2\sqrt{\left(2l-\Delta h\right)\Delta h}$, where ∆h is the maximum height difference within a step. The average speed estimation is obtained by dividing the sum of step lengths by the sum of step durations, and then multiplying by an empirical factor of 1.25 as $v=1.25(\sum s_{i}/\sum t_{i})$ [7]. The method that we evaluated was similar to the one used in [13], which estimates the length of each half step as $1.25\sqrt{\left(2l-\Delta h\right)\Delta h}$, where ∆h is the absolute peak-to-peak height change. We now estimated the peak-to-peak height change as the absolute difference between each peak and the average of the two adjacent peaks with an opposite sign; therefore, per step, we estimated two half step lengths, one for a negative and one for a positive peak. The height signal was estimated as the second integral of the vertical acceleration, which was estimated by the acceleration norm. We high pass filtered the signal forward and backward at each process of integration to derive the height from acceleration (i.e., as accelerations, velocities, and heights) with a second order Butterworth filter with a cut-off frequency of 0.5 Hz. The cut-off frequency was increased from 0.1 Hz in the paper from Zijlstra and Hoff [7] to 0.5 Hz, in order to further limit effects of integration drift that may lead to large errors in height differences. This may be especially relevant when using a pendant, because of short bounces. The cut-off frequency was limited to 0.5 Hz to stay below realistic step frequencies.

**Figure 2:**
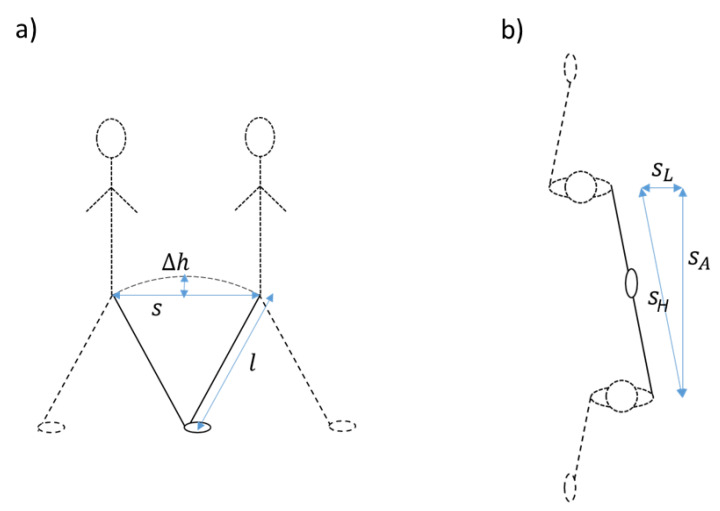
Explanation of the inverted pendulum model ((**a**), side view) and of the addition of lateral movement in our updated version of the algorithm ((**b**), top view).

**Figure 3:**
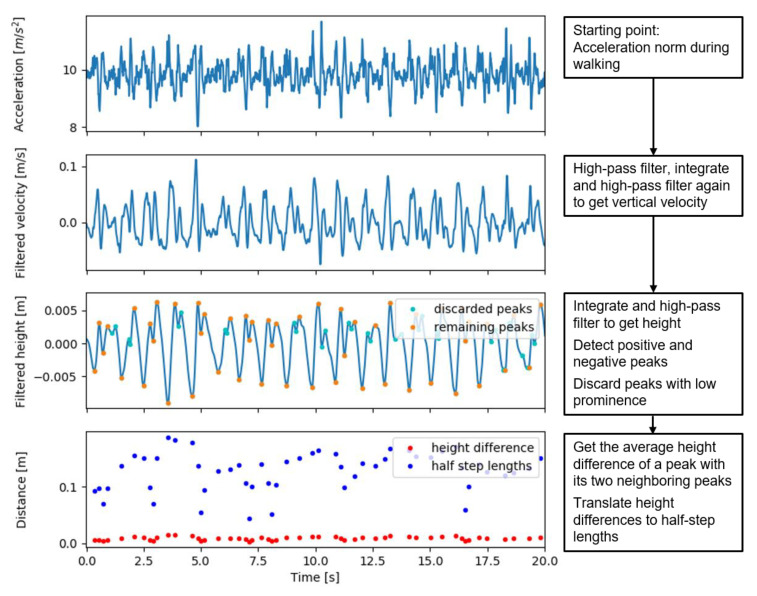
Acceleration, velocity, and height signals with detected peaks in the different steps of the updated inverted pendulum walking speed estimation algorithm.

We updated the method to account for lateral movements in the step trajectory in order to improve estimates for slow walking speeds. First, step lengths become small in slow walking [14], which makes the effect of lateral movement on the height changes more prominent. To correct for this, we assumed that the circular trajectory of the CoM during a step consists, horizontally, of an anterior and a lateral component, the Euclidian length of which, or the horizontal step length, determines the height change. Thus, in order to account for the lateral movement, we subtracted an estimate of the distance of lateral movement from the estimated horizontal step length in a quadratic way as $s_{A}=\sqrt{s_{H}{}^{2}-s_{L}{}^{2}}$, where $s_{A}$ is the anterior (or forward step length) and $s_{H}$ is the horizontal step length. The lateral movement $s_{L}$ was estimated as 5.4° or 0.094 times the leg length *l* [15]. Figure 2 shows the variables that were used in the IP model and in the updated model, including the addition of lateral movement (Figure 2b), and Figure 3 shows the signals at the different stages of the algorithm. 

Secondly, due to movement variability, additional peaks may disturb the limited height changes in slow walking, leading to more than one positive and one negative height peak appearing per step. The additional peaks, even if they are small, can substantially increase the summed step lengths, due to the non-linear relation between height change and step length. Therefore, we filtered peaks by a minimum prominence. The threshold for the minimum prominence was set to $\Delta h_{p_{min}}=l-\sqrt{l^{2}-{\left(s_{L}/1.25\right)}^{2}}$, which is equal to a height change that corresponds to a forward step length $s_{A}=0$, given the subtraction of the estimated lateral movement distance $s_{L}$. 

### 2.4. Analyses

The mean speed for each walking activity was estimated and compared to the reference speed. As validation statistics, we used Pearson correlation (R), root mean square error (RMSE), and mean absolute error (MAE) per study and device location combination. For Simulated Hospital, the results for Treadmill, 6MWT, and Walking Aids were separately determined. We also determined the statistics for all data combined, and for all data from speeds <0.5 m/s as well as for all data from speeds ≥0.5 m/s. 

For the (absolute/ squared) errors, we determined the *p*-values to determine whether the results for the updated IP method were statistically significantly different (*p* < 0.01) from the IP method for the same data. This was done using Wilcoxon signed-rank tests, as the differences were not normally distributed (Shapiro–Wilk test for normality, *p* < 0.05). To compare the results between wearing positions, we selected the subset of data for which measurements were available for all three sensor locations and determined MAE per wearing position for all of these data combined, for the data from speeds < 0.5 m/s as well as for the data from speeds ≥ 0.5 m/s. For these MAEs, we calculated the *p*-values using Wilcoxon signed-rank tests for comparisons between the different positions.

## 3. Results

### 3.1. Results for Both Algorithms, Per Activity

**Figure 4:**
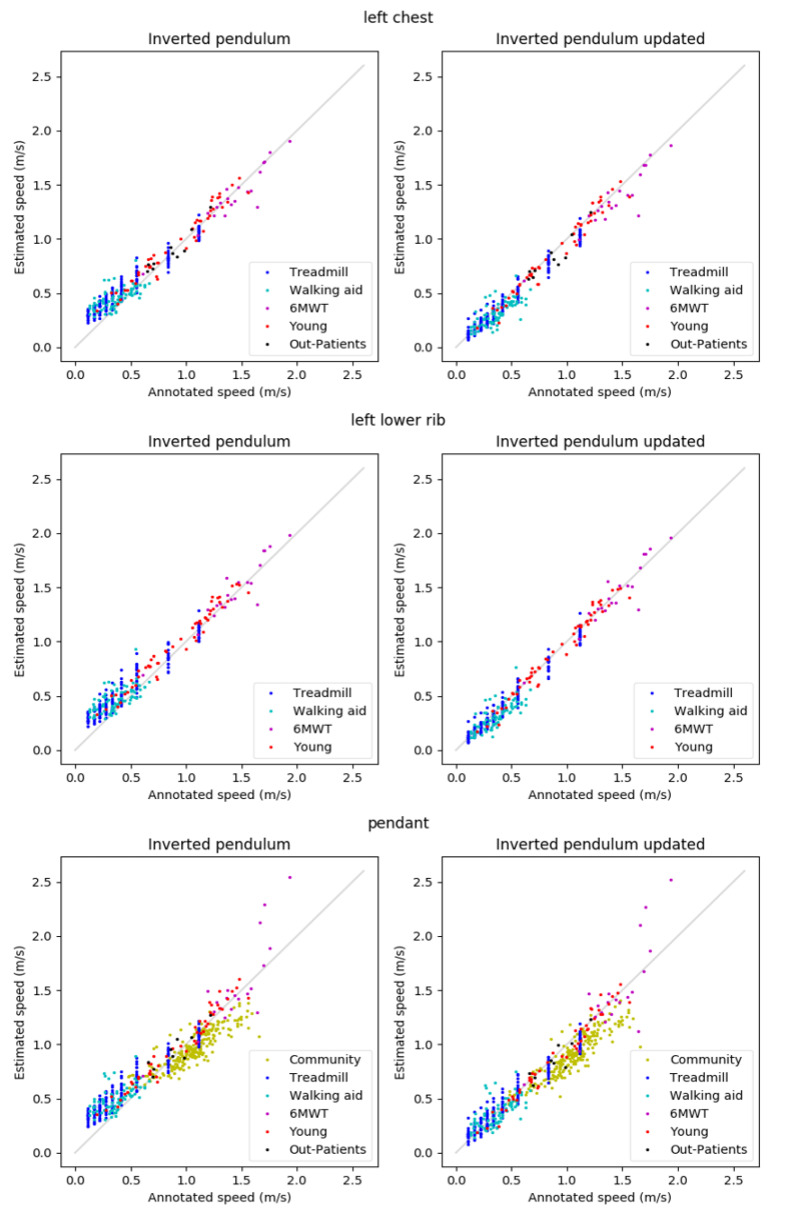
Results for the left chest (**top**), left lower rib (**middle**), and pendant (**bottom**) locations.

The reference walking speeds ranged from 0.11–1.93 m/s. The scatterplots shown in Figure 4 show that estimations are typically well in line with the annotations. Exceptions are the overestimations of low speeds for the basic IP method, the underestimation of the speeds in Community for both methods, and the estimation errors for the speeds above 1.6 m/s in 6MWT for the pendant position. 

Table 2 presents the algorithm performance metrics (R, RMSE, and MAE). For the IP method, Pearson correlations were > 0.84 for the Community, Treadmill, 6MWT, Young, and Out-Patients data, and 0.60–0.67 for Walking aid data. Generally, the updated IP method resulted in equal or stronger Pearson correlations as compared to the original IP method, although the results per activity varied. The results for all data combined were better for the updated IP method, with most improvement in the lower walking speeds. When looking at wearing position, for both algorithms the Pearson correlations were higher for the two body fixed locations as compared to the pendant location.

The RMSE and MAE show similar results, with RMSE and MAE being quite close. At speeds that are below 0.5 m/s, for the IP method, RMSE and MAE were 0.15–0.19 m/s and 0.13–0.17 m/s, respectively, and, at higher speeds, this was 0.09–15 m/s (RMSE) and 0.07–0.11 m/s (MAE). At low speeds, the results improved significantly (*p* < 0.01) with the updated IP method for all wearing positions, leading to RMSEs and MAEs of 0.06–0.09 m/s and 0.05–0.06 m/s, respectively. At higher speeds, for the pendant location MAE increased slightly, but significantly (*p* < 0.01) with the updated method, from 0.11 m/s to 0.13 m/s, whereas the results for the other wearing positions did not change significantly. For all the data combined, the results were better for the updated algorithm for all three wearing positions, with a higher correlation coefficient, and statistically significantly lower RMSE and MAE (*p* < 0.01) when compared to the IP method. 

### 3.2. Comparisons between Wearing Positions

Table 3 contains the Pearson correlation coefficients, RMSEs and MAEs per wearing position for both methods for the data points that were available for all three wearing positions. This means that we excluded the Community and Out-patients data as well as data from two participants of the Young dataset. For both of the speed ranges, the Pearson correlation coefficient is the lowest for the pendant location, for both algorithms. For speeds that are below 0.5 m/s, the absolute errors for the fixed wearing positions (chest and rib) are lower (*p* < 0.01) than for the pendant, whereas, for higher speeds, the only statistically significant difference is that between the rib and chest location for the updated method, with lower absolute errors for the rib position (*p* < 0.01). For all of the data combined, with the IP method the chest position was superior to the rib position, which was, in turn, better than the pendant position (*p* < 0.01), but, with the updated IP method, this superiority of the chest position disappeared.

## 4. Discussion

### 4.1. Findings

The original IP walking speed estimation method, as used by Rispens et al. [13], was evaluated for new wearing locations and at low walking speed, as found in the older and patient populations. For these low walking speeds, the IP method was further improved by accounting for lateral movement and for the occurrence of more than one peak per step in the height signal. In general, both of the methods provided estimations that were in line with reference speeds for the new wearing locations. However, the original IP method did result in an overestimation of speed for slow walking, which was successfully counteracted with the updated IP method. 

At the higher walking speeds of above 1.6 m/s that we measured in the 6MWT in healthy volunteers, the spread as compared to reference speeds was larger for both the original and updated IP method for the pendant position, which resulted in lower accuracies. This could be due to the swinging of the pendant at these higher speeds. While this is something to keep in mind when choosing a wearing position and algorithm for walking speed monitoring for a specific application, walking speeds that are above 1.6 m/s may not be relevant for monitoring health in older community and patient populations. Nevertheless, it should be noted that, also for the Community data from older people—for which only data at the pendant location were available—the accuracy was lower when compared to other normal (i.e., without walking aids) activities, with an underestimation bias for both methods. 

### 4.2. Context

Our results that were generated with the IP model are in line with those of Zijlstra and Hof [7], even though they were obtained from different wearing positions. In the speed range of ~0.5–2.0 m/s, Zijlstra and Hof [7] reported RMSEs from 0.05–0.14 m/s, where we obtained 0.09–0.15 m/s, depending on the wearing position. Another interesting study for comparison is the one from Byun et al. [16], who investigated the walking speed estimations from a sensor at the lower back on a large group (n = 659) of older Korean adults and compared the results from their regression model to those that were obtained with the model of Zijlstra and Hof. With their regression model, they obtained an RMSE of 0.07 m/s, which was better than the RMSE of 0.26 m/s that they obtained for the IP model [16]. It is not clear why their RMSE for the IP model appears to be higher than that reported by Zijlstra and Hof, as the tested speed range of ~0.6–1.7 m/s is comparable. Overall, the regression model of Byun et al. also has a lower RMSE when compared to our updated IP model for the > 0.5 m/s speed range (0.07 m/s as compared to 0.09–0.13 m/s, depending on wearing position). However, a problem with regression models is that they often lack generalizability. Although Byun et al. studied a large cohort, only the elderly were included, which were walking over a flat straight walkway. Additionally, because no walking speeds below 0.5 m/s were included, this model may not work for those relevant lower speed ranges, where walking patterns may become different. With our results, we showed that the biomechanical-based updated IP model is robust for different wearing positions, populations, walking activities, and walking speeds, which makes it applicable for various use cases.

### 4.3. Implications

Advances in wearable sensing technologies have driven recent research towards automated falls detection and the continuous telemonitoring of physical activity, both at home and in the hospital. Our new method allows for a more accurate measurement of the walking speed in frail populations, which can provide a valuable tool for monitoring the state of health of patients and older people in the community [17]. 

### 4.4. Limitations

Although patients and older people were included in the study data, the very low walking speeds below 0.5 m/s were simulations from individuals with much higher preferred walking speeds. The method might perform differently for patients and older people that truly have such very low preferred walking speeds. In addition, the effect of severe pathological gait on the walking speed estimate accuracies may deviate from what we found here. This would need to be further validated in the targeted patient groups. 

### 4.5. Future Work

Apart from monitoring walking speed, the estimation method that is assessed here could enable the monitoring of total distance covered in older people and patients. Both of these purposes will require a walking detection method, to select the walking periods for the determination of average walking speed and distance walked in home or hospital settings. The accuracy of such estimates needs further assessment, especially in the relevant home and hospital settings in the target populations.

## 5. Conclusions

We showed that walking speed estimations that are based on the IP method are also valid when applied to pendant, chest, and lower rib wearing positions. The updated IP method improved accuracy at the low walking speeds when compared to the original IP method, while giving similar results for higher walking speeds. When comparing the results for different wearing positions, for this updated algorithm MAEs were very close to each other, which indicated that all three wearing positions could be suitable for speed estimations. Overall, the method proved to be applicable under a variety of walking conditions, and in a wide range of individuals, proving its robustness for variations in walking patterns.

## Figures and Tables

**Table 1:** Demographics of the included studies. Age, height, and weight are presented as mean ±SD.

	N	Female/Male	Age (y)	Height (m)	Weight (kg)
Young	9	1/8	35.0 ± 4.6	1.83 ± 0.06	73.9 ± 7.5
Out-Patients	10	5/5	73.0 ± 4.7	1.71 ± 0.09	89.2 ± 20.2
Simulated Hospital	20	10/10	43.3 ± 13.4	1.75 ± 0.11	78.4 ± 14.8
Community	120	82/38	80.4 ± 6.7	1.63 ± 0.09	71.1 ± 14.8

**Table 2:** Pearson correlation Pearson correlation (R), root mean square error (RMSE), and mean absolute error (MAE) for the three locations and two methods. The lower nine rows show the results for the combined data for speeds below 0.5 m/s, for speeds ≥0.5 m/s and for all data combined. * Indicates that the Wilcoxon signed-rank test showed that the underlying absolute errors were statistically significantly lower for this algorithm compared to those for the other one, with *p* < 0.01.

		Chest		Rib		Pendant	
		IP	Updated IP	IP	Updated IP	IP	Updated IP
Community	R	-	-	-	-	0.86	0.88
	RMSE [m/s]	-	-	-	-	0.15 *	0.18
	MAE [m/s]	-	-	-	-	0.13 *	0.16
Treadmill	R	0.97	0.98	0.96	0.98	0.96	0.97
	RMSE [m/s]	0.13	0.06 *	0.14	0.06 *	0.17	0.08 *
	MAE [m/s]	0.12	0.05 *	0.12	0.05 *	0.15	0.06 *
Walking aid	R	0.66	0.75	0.64	0.78	0.60	0.64
	RMSE [m/s]	0.14	0.10	0.15	0.10 *	0.18	0.11 *
	MAE [m/s]	0.11	0.08	0.12	0.08 *	0.14	0.09 *
6MWT	R	0.94	0.93	0.94	0.94	0.84	0.82
	RMSE [m/s]	0.10	0.13	0.11	0.11	0.25	0.26
	MAE [m/s]	0.07	0.08	0.08	0.08	0.17	0.17
Young	R	0.98	0.98	0.98	0.99	0.98	0.98
	RMSE [m/s]	0.09	0.07 *	0.10	0.06 *	0.10	0.07 *
	MAE [m/s]	0.08	0.05 *	0.09	0.05 *	0.09	0.05 *
Out-Patients	R	0.93	0.93	-	-	0.90	0.90
	RMSE [m/s]	0.07	0.08	-	-	0.09	0.08
	MAE [m/s]	0.06	0.05	-	-	0.08	0.05
<0.5 m/s	R	0.72	0.81	0.70	0.82	0.67	0.73
	RMSE [m/s]	0.15	0.07 *	0.16	0.06 *	0.19	0.09 *
	MAE [m/s]	0.13	0.05 *	0.14	0.05 *	0.17	0.06 *
≥ 0.5 m/s	R	0.97	0.97	0.96	0.98	0.87	0.90
	RMSE [m/s]	0.09	0.10	0.10	0.09	0.15 *	0.16
	MAE [m/s]	0.07	0.07	0.08	0.07	0.11 *	0.13
All	R	0.98	0.98	0.97	0.98	0.94	0.95
	RMSE [m/s]	0.13	0.08 *	0.14	0.08 *	0.16	0.14 *
	MAE [m/s]	0.10	0.06 *	0.11	0.06 *	0.13	0.10 *

**Table 3:** Pearson correlation R, MAE, and RMSE for the three locations and two methods, for the subset of data that was available for all three wearing positions. ^a^ indicates that the Wilcoxon signed-rank test showed that the underlying absolute errors were lower for this wearing position compared to those for the left lower rib wearing position, with *p* < 0.01, ^b^ indicates that the Wilcoxon signed-rank test showed that the underlying absolute errors were lower for this wearing position compared to those for the pendant wearing position, with *p* < 0.01, and ^c^ indicates that the Wilcoxon signed-rank test showed that the underlying absolute errors were lower for this wearing position when compared to those for the chest wearing position, with *p* < 0.01.

		IP			Updated IP		
		Chest	Rib	Pendant	Chest	Rib	Pendant
<0.5 m/s	R	0.72	0.70	0.65	0.81	0.82	0.72
	RMSE [m/s]	**0.15 ^a,b^**	**0.16 ^b^**	0.19	0.07	0.07	0.09
	MAE [m/s]	**0.13 ^a,b^**	**0.14 ^b^**	0.17	0.05	0.05	0.06
≥0.5 m/s	R	0.97	0.96	0.94	0.97	0.98	0.95
	RMSE [m/s]	0.09	0.10	0.14	0.10	**0.09 ^c^**	0.13
	MAE [m/s]	0.07	0.08	0.10	0.08	**0.07 ^c^**	0.09
all	R	0.98	0.97	0.96	0.98	0.98	0.96
	RMSE [m/s]	**0.13 ^a,b^**	**0.14 ^b^**	0.17	0.08	**0.08 ^c^**	0.11
	MAE [m/s]	**0.11 ^a,b^**	**0.11 ^b^**	0.14	0.06	**0.06 ^c^**	0.07

## Data Availability

Informed consent obtained from the subjects involved in the studies does not cover publicly sharing the data. Data may be available upon request at discretion of the authors and pending approvals.
